# Metastatic mesenchymal chondrosarcoma of the spine managed with nonsurgical treatment: A case report and review of the literature

**DOI:** 10.1002/ccr3.6712

**Published:** 2022-12-09

**Authors:** Leeann Qubain, Brandon P. Hirsch, Naresh Reddivalla, Ali A. Baaj, Lee R. Leddy, Robert A. Ravinsky

**Affiliations:** ^1^ University of Arizona College of Medicine – Phoenix Phoenix Arizona USA; ^2^ Department of Orthopaedics Surgery University of Arizona College of Medicine – Phoenix Phoenix Arizona USA; ^3^ Banner Desert Medical Center Mesa Arizona USA; ^4^ Department of Neurosurgery University of Arizona College of Medicine – Phoenix Phoenix Arizona USA; ^5^ Department of Orthopaedics and Physical Medicine Medical University of South Carolina Charleston South Carolina USA

**Keywords:** chemotherapy, mesenchymal chondrosarcoma, metastatic disease, radiation, spine, surgical resection

## Abstract

In this report, we present a rare case of a 17‐year‐old male patient with metastatic mesenchymal chondrosarcoma (MCS) managed with nonsurgical treatment who subsequently demonstrated a favorable response to concurrent chemotherapy and radiation therapy, followed with pazopanib target therapy. Further study regarding nonoperative care for metastatic MCS of spine is warranted.

## Abstract 2

Although surgical treatment has been recommended for MCS, this case report illustrates that metastatic MCS of the spine may be effectively managed nonsurgically, obviating the morbidity of surgical intervention.

## 1 INTRODUCTION

Mesenchymal chondrosarcoma (MCS) is a rare, aggressive form of extraosseous chondrosarcoma, representing <1% of all chondrosarcomas.^1^ MCS most commonly affects the craniofacial bones, ribs, and spine, particularly the thoracic spine.^2^, ^3^ MCS seems to affect all age groups, but there is a peak incidence in the second decade of life and slight predilection for females.^2^ Prognosis is unpredictable, but overall survival is poor with an estimated 10‐year survival rate of <30%.^1^, ^3^ Nussbeck et al.^1^ reviewed 10 cases and proposed that proliferative activity may be a useful parameter to predict prognosis for patients with MCS. Amer et al.^4^ performed a retrospective review of the Surveillance, Epidemiology and End Results Program (SEER) database to evaluate prognosis of nonconventional chondrosarcoma subtypes and found that patients with MCS had a median survival of 33.5 months and 5‐year survival rate of 51%.^5^

Because MCS of the spine is exceptionally rare, there is no standardized treatment regimen or protocol. It is generally agreed upon that the role of surgery is limited in metastatic MCS. However, published case series and case reports have suggested that surgery is indicated in the setting of neoplastic instability or neurological compression in patients with metastatic MCS of the spine causing mechanical axial pain, radicular symptoms, or neurological deficits.^6^ The choice of surgical approach and surgical strategy depends on a variety of factors including spinal stability, neurological status, the presence or absence of high‐grade epidural disease, patient performance status, prognosis, and consideration of possible adjuvant chemotherapy or radiation therapy.^7^ With respect to surgical treatment, it has been observed that there is a direct correlation with the extent of surgical resection and patient survival.^8^ In a systematic review including 107 patients, Xu et al.^9^ concluded that surgery is necessary in management of MCS in bone and soft tissue. In a series of 113 patients with MCS, Frezza et al.^5^ observed that in patients with localized disease who underwent surgical resection, local recurrence rates were lowest at 2% after en bloc resection with negative margins compared to local recurrence of 27% in patients who underwent resection with positive margins. However, achieving negative margins for en bloc resection for a large primary tumor can be technically difficult to achieve, and may not always be feasible, particularly in the spine. Seventeen of the patients from the study by Frezza et al. had a metastatic MCS diagnosis, of which only two patients survived who were treated with combination radiotherapy and chemotherapy. One of those patients was a 14‐year‐old boy with pelvic MCS metastatic disease managed with radiotherapy and chemotherapy who was progression‐free for 8 years.^5^ The role of adjuvant radiotherapy and chemotherapy for the treatment of MCS is debated, as some studies note that MCS is resistant to these modalities, while others have reported favorable outcomes.^10^ Although surgical treatment has been previously recommended for MCS, this case report illustrates that metastatic MCS of the spine may be effectively managed nonsurgically, obviating the morbidity of surgical intervention.

## 2 CASE PRESENTATION

### 2.1 Initial presentation

A 17‐year‐old male patient presented initially to urgent care with a chief complaint of axial mechanical low back pain of 3 months duration that subsequently progressed to affect the upper/mid back as well. Over this time period, the patient noted 15 pounds of unexplained weight loss. There were no complaints of gait imbalance, loss of dexterity, weakness, paresthesias, or sphincter dysfunction. He was neurologically intact. Urgent care evaluation included plain radiography demonstrating lytic lesions throughout the thoracolumbar spine and multiple compression fractures. The patient was then referred to spine surgery and medical oncology. Initial evaluation by spine surgery took place in July 2020.

### 2.2 Investigations

**FIGURE 1:**
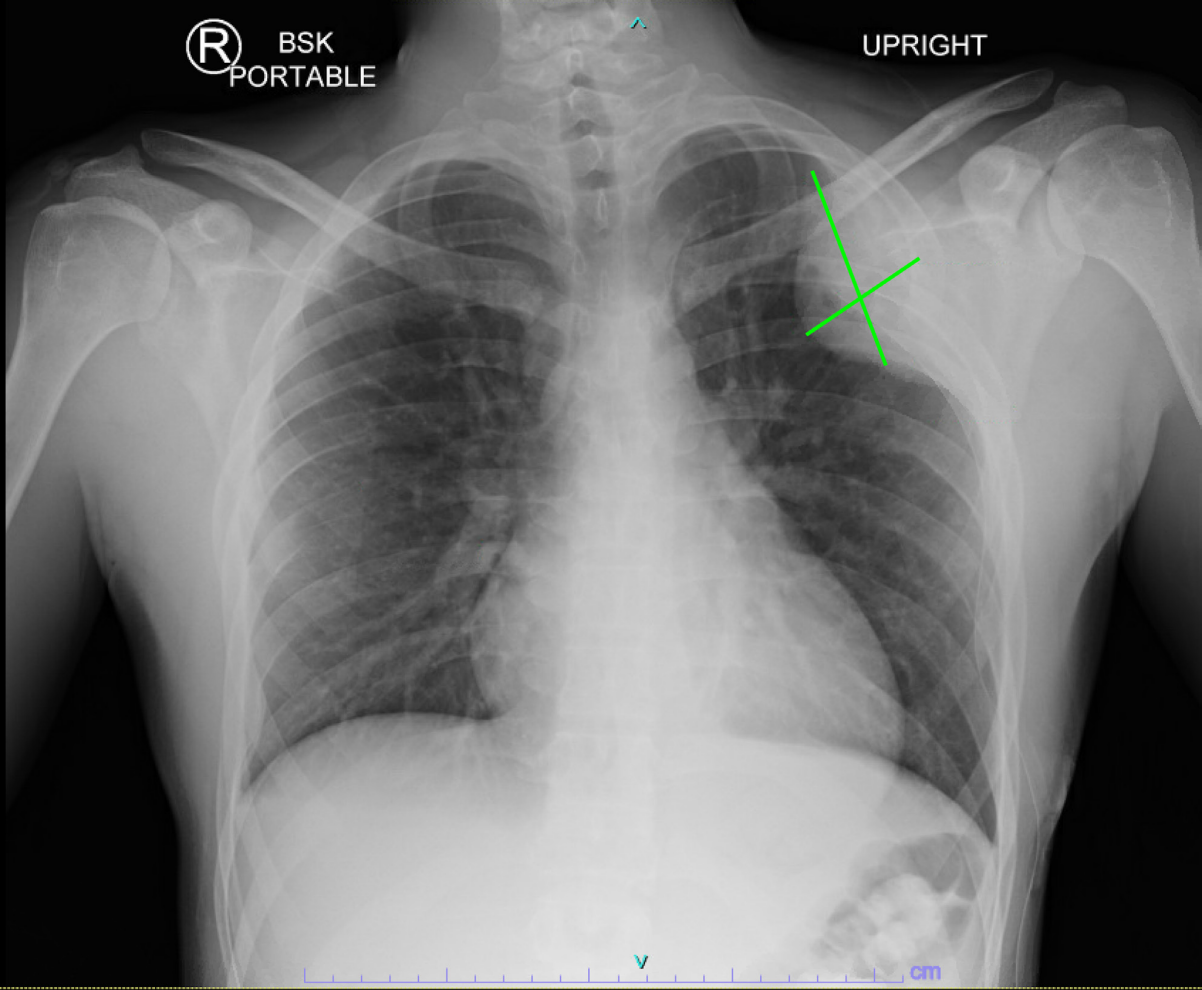
Pre‐treatment chest radiograph showing a large, left‐sided rib lesion measuring 4.49 cm × 7.98 cm. Multiple additional lytic lesions can be seen in the ribs.

**FIGURE 2:**
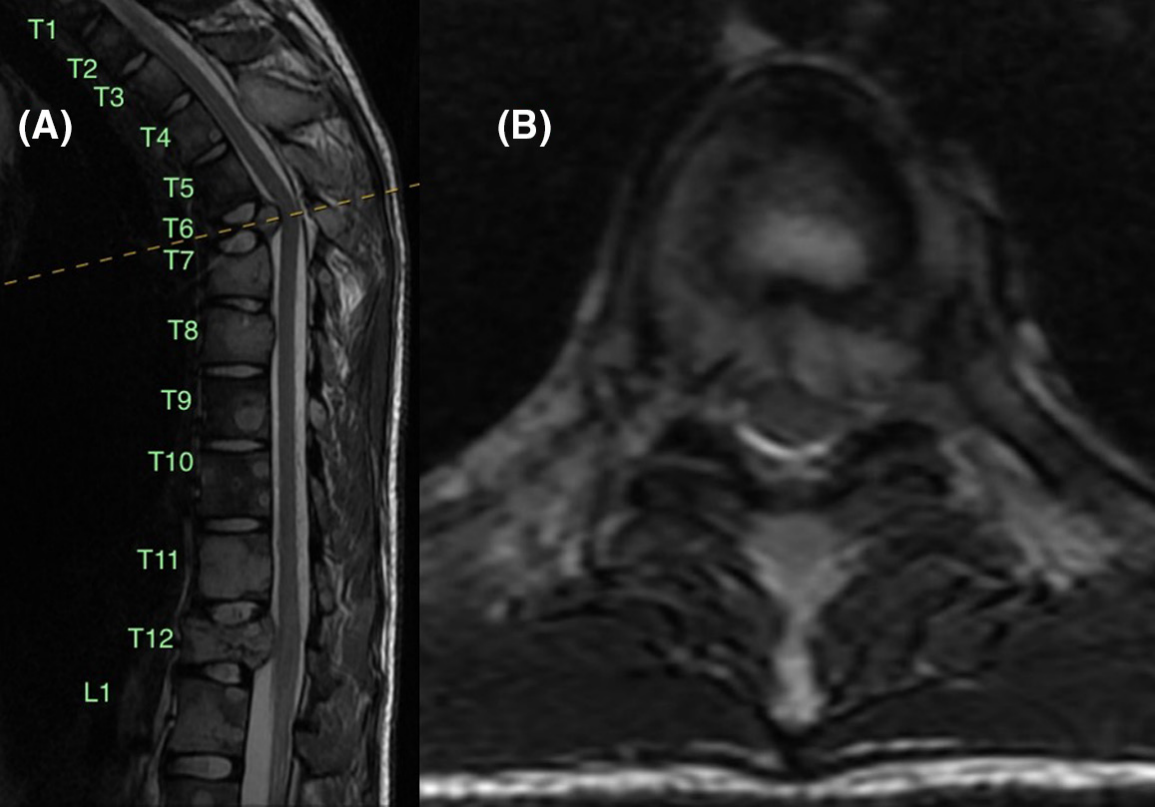
Pretreatment MRI of the thoracic spine. (A) Mid‐sagittal T2‐weighted image demonstrates diffuse, multifocal bone lesions through the thoracic and rostral lumbar spine. Pathologic fractures seen at T6 and T12 in this image. (B) Axial T2‐weighted image at the level of the T6 vertebral body shows central canal stenosis and high‐grade CSF effacement with spinal cord deformation in the absence of signal change.

**FIGURE 3:**
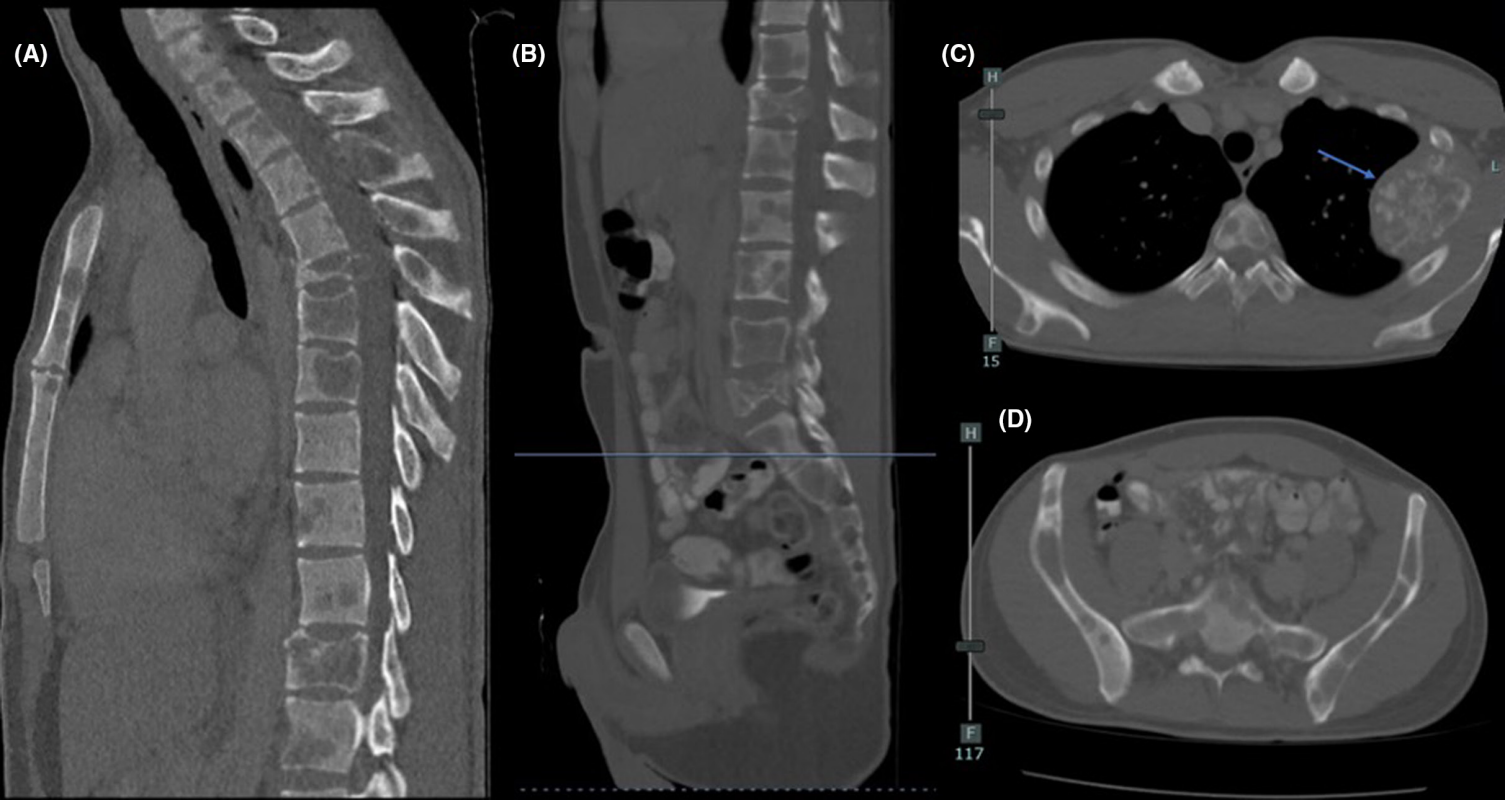
Selected pre‐treatment CT images, including mid‐sagittal images of the thoracic (A) and lumbar spine (B), and selected axial images in the thoracic spine (C) at the level of the left fourth rib, where the presumed primary lesion was localized (blue arrow), as well as at the lumbosacral junction/pelvis (D). On the sagittal images, pathologic fractures can be seen at T6, T12, and L5, and on all images multiple lytic lesions can be appreciated throughout the thoracolumbar spine and pelvis.

**FIGURE 4:**
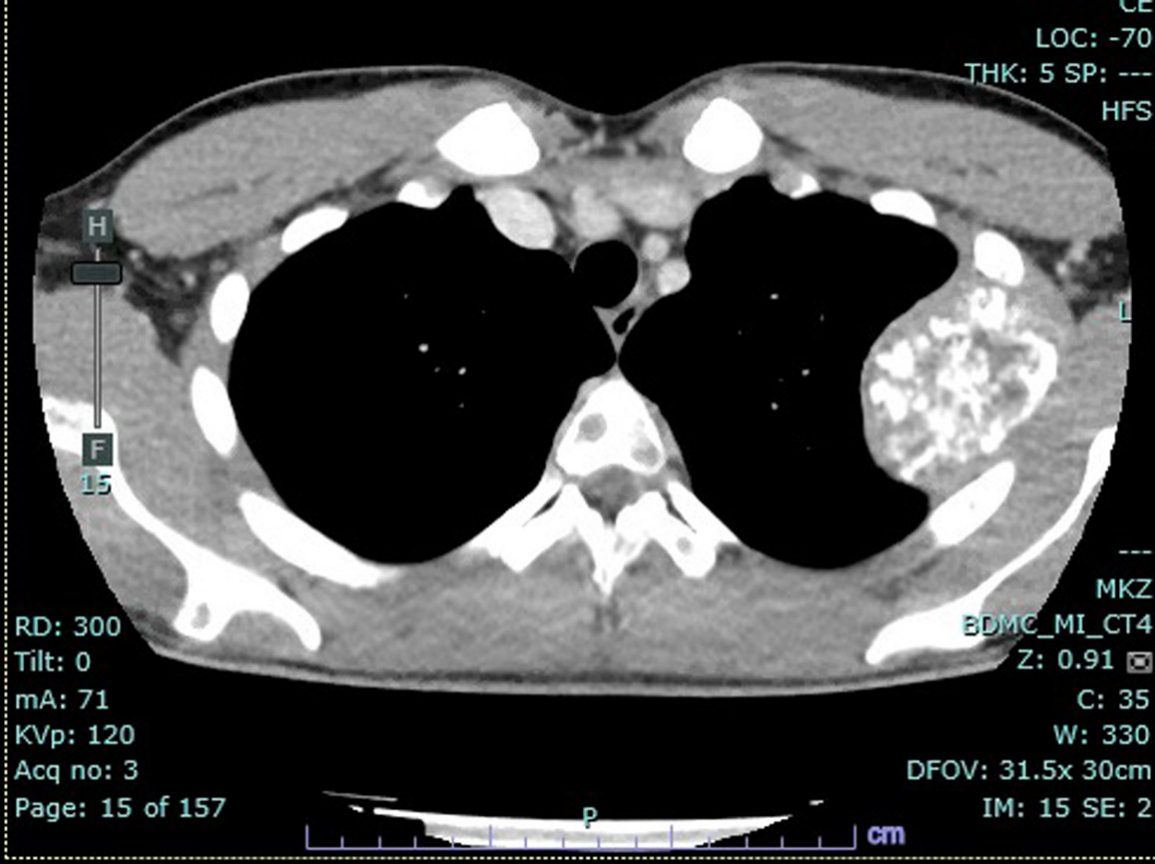
Selected pre‐treatment CT chest axial image demonstrating the presumptive primary lesion, located at the left fourth rib and extending into the thoracic cavity. This lesion demonstrates the typical radiologic features of “popcorn calcifications” characteristic of chondrosarcoma. Secondary metastatic lesions can also be seen within the vertebrae and the right scapula. CT chest/abdomen/pelvis scan did not show evidence of significant visceral metastatic lesions, including lesions of the soft tissues and lymph nodes, the liver and lungs.

**FIGURE 5:**
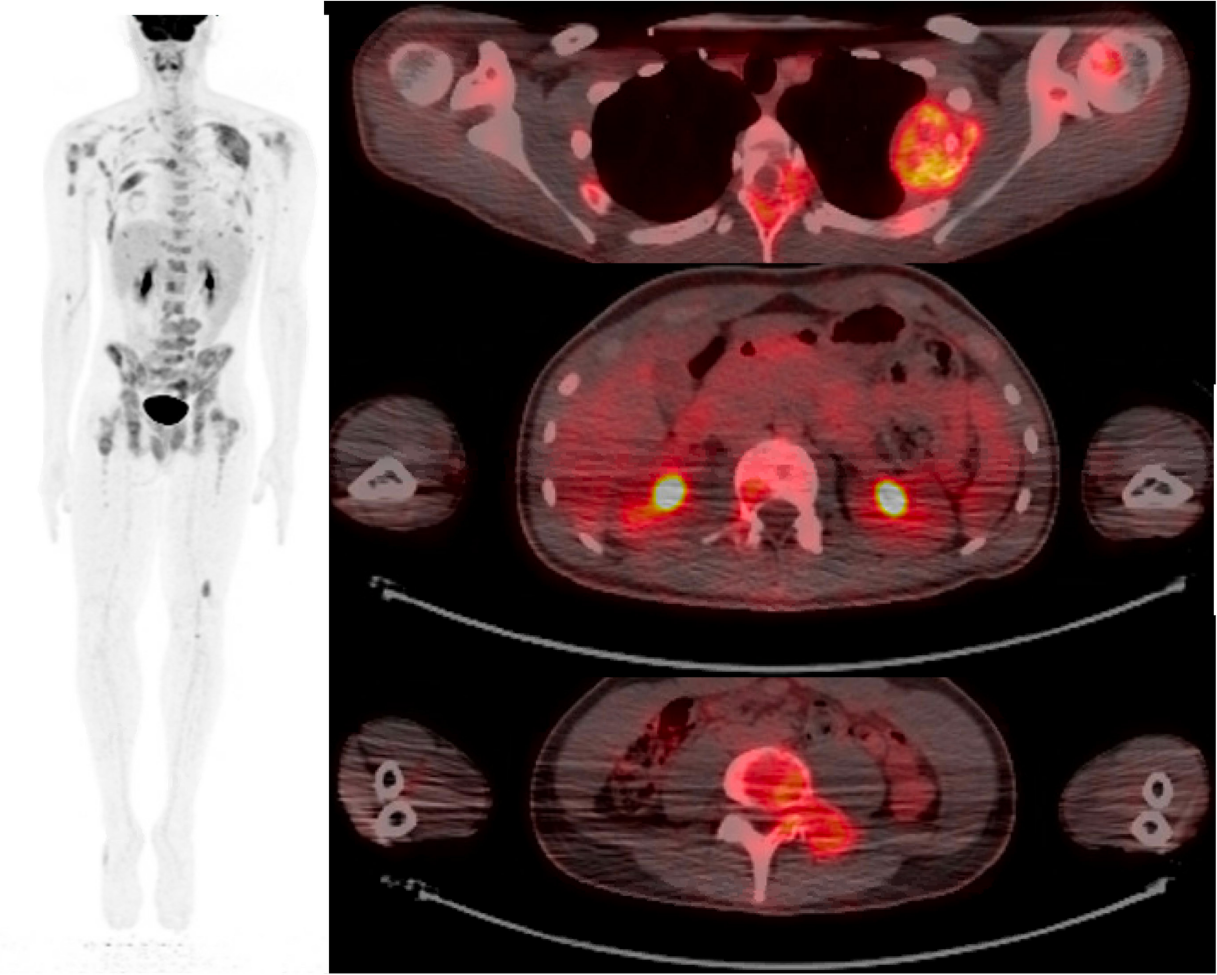
Pre‐treatment PET revealed a large, hypermetabolic mass of the left, lateral fourth rib as well as numerous smaller lesions throughout the axial and appendicular skeleton. PET scan did not show evidence of significant visceral metastatic lesions, including lesions of the soft tissues, lymph nodes, liver, and lungs.

**FIGURE 6:**
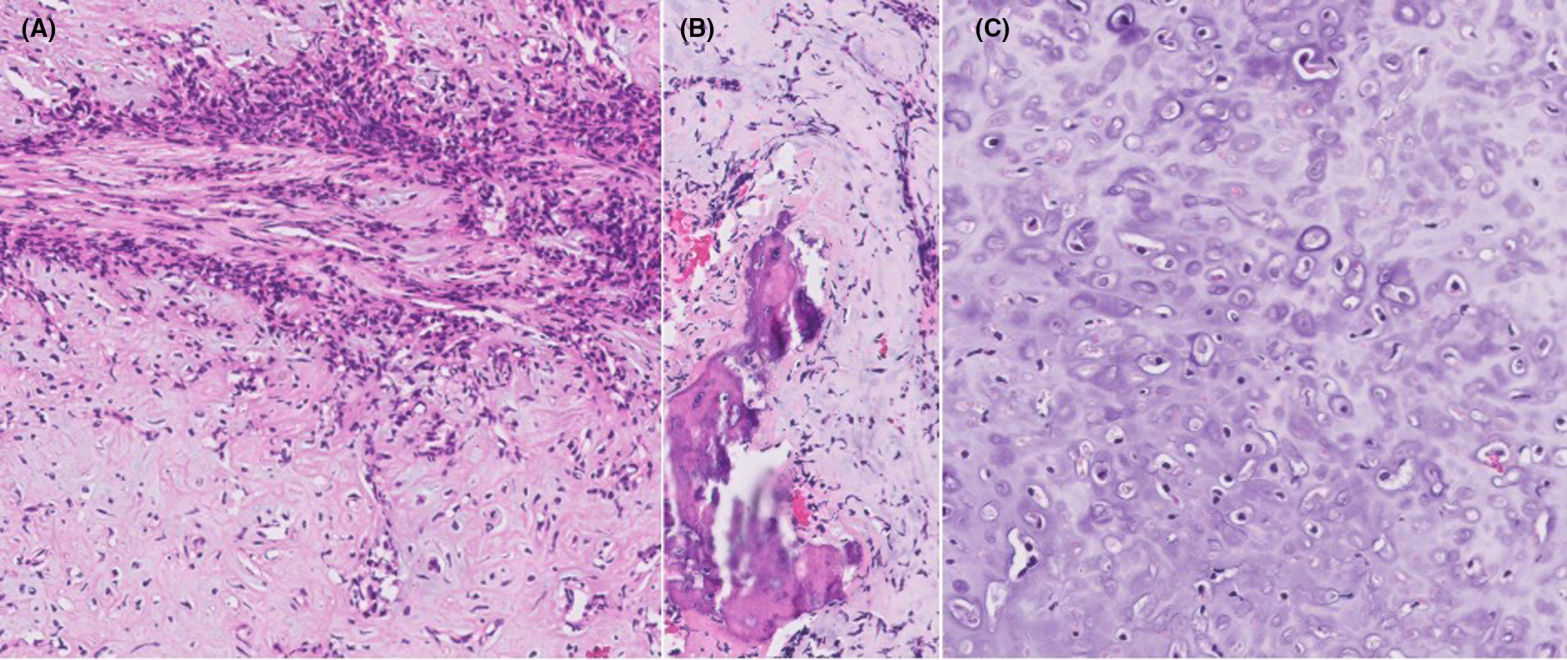
(A) Pathology from chest lesion biopsy specimen. Shows biphasic tumor with focal areas of calcification. (B) Shows mix with small round cells, arranged in sheets, clusters, and trabecular‐like growth. (C) Evidence of high‐grade malignancy. This described histology for our patient is consistent with characteristics of MCS, described as a biphasic pattern of undifferentiated small blue, round cells with islands of hyaline cartilage.^5^

Local and systemic investigations were carried out, including repeat chest radiographs (Figure 1), full spine magnetic resonance imaging (MRI), computed tomography (CT), and positron emission tomography (PET). MRI of thoracic and lumbar spine showed diffuse, multifocal bone lesions through the thoracolumbar spine, sacrum, and pelvis. Pathologic compression fractures were present at T6, T12, and L5, and there was central canal stenosis at the level of the T6 vertebral body without complete CSF effacement or signal change within the spinal cord (Figure 2). CT chest/abdomen/pelvis showed extensive, destructive lytic bone lesions throughout the chest including in multiple ribs, multiple vertebrae, the sternum, bilateral scapulae, and the pelvis (Figure 3). The largest lesion appeared on the lateral portion of the left fourth rib, measuring approximately 6.6 × 6.1 × 5.7 cm (Figure 4). CT and PET scans did not show evidence of significant visceral disease involving the lymph nodes, liver, or lungs. PET scan revealed a hypermetabolic mass of the left lateral fourth rib, consistent with chondrosarcoma, as well as hypermetabolic lesions throughout the axial and appendicular skeleton (Figure 5). The left fourth rib mass was the suspected primary lesion, while the other lesions were believed to be metastases. Open biopsy of the left fourth rib lesion provided specimens for pathology (Figure 6). This showed evidence of a biphasic tumor admixed with small round cells and focal areas of calcifications. Gene analysis with RNA sequencing revealed that the lesion contained a fusion of HEY‐1‐NCOA2, consistent with MCS.

### 2.3 Initial management

**FIGURE 7:**
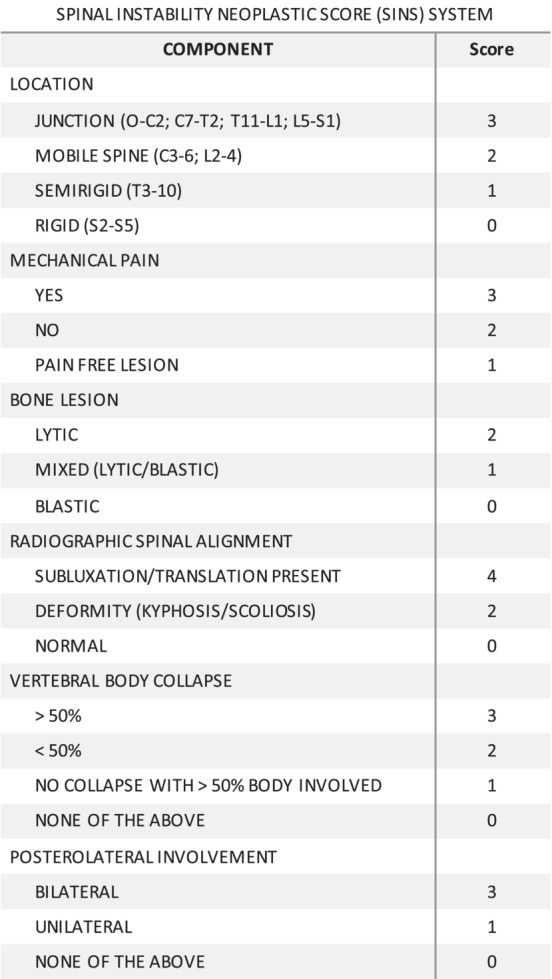
The spinal instability neoplastic score (SINS).^15^ This score can range from 0 to 18 and determines the spinal instability of patients with neoplastic lesions of the spine. A score from 1 to 6 indicates a stable spine, while a score from 7 to 12 is potentially unstable, and a score ranging from 13 to 18 is considered to be unstable.^15^

The spinal instability neoplastic score (SINS) for our patient was 18, suggesting a lack of spinal stability (Figure 7). However, the diffuse presence of osseous metastatic lesions throughout the thoracolumbar spine rendered the possibility of a focal stabilizing procedure prohibitively difficult. Moreover, the patient was not clinically myelopathic, and there was no signal change within the spinal cord on MRI. For these reasons, a multidisciplinary decision was made to treat the patient initially with radiation, chemotherapy, and TLSO bracing. Further discussion with pathology and medical oncology lead to a combined decision to utilize VDC/IE chemotherapy and proton beam radiation, a Ewing's sarcoma‐based treatment regimen.^11^ VDC/IE therapy, which was initiated in April 2021, consists of alternating chemotherapy every 2–3 weeks between two medication combinations. The first combination is comprised of vincristine, doxorubicin, and cyclophosphamide, and the second is comprised of ifosamide and etoposide. This contrasts with traditional dogma, as chondrosarcoma is typically resistant to chemotherapy and therefore treated with wide surgical excision when possible.

Following VDC/IE chemotherapy regimen, the patient underwent two cycles of oral pazopanib. The patient tolerated the treatment regimen well.

The patient additionally underwent radiation therapy for 3 months, which consisted of proton beam radiation to his chest and pelvis. He received 4500 cGy in 25 fractions at 180 cGy per fraction to his chest and pelvis. He received additional radiation, which included two pelvis boosts to a total dose of 540 and 360 cGy in 3 and 2 fractions at a dose of 180 cGY per fraction and a chest boost total dose 1980 cGY in 11 fractions at a dose of 180 cGY per fraction.

### 2.4 Follow‐up

**FIGURE 8:**
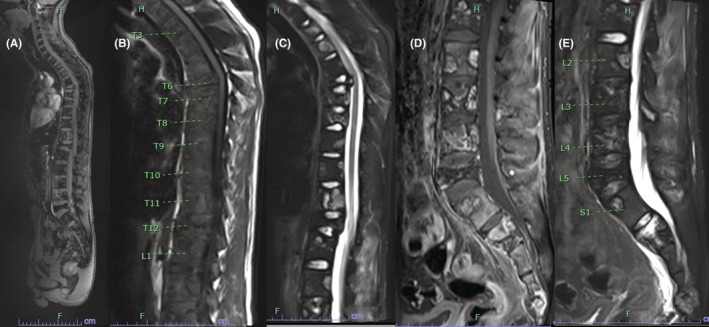
Post‐treatment MRI, including the sagittal localizer (A), T1‐ (B) and T2‐weighted fat suppression sequence (C) of the thoracic spine, and T1‐ (D) and T2‐weighted sequence (E) of the lumbar spine 10 months after the patient‐initiated treatment with VDC/IE chemotherapy and proton beam radiation treatment. These images demonstrate improvement in the size of the osseous lesions with resolution of epidural disease.

Imaging 10 months after initiation of the VDC/IE chemotherapy regimen, in February 2022, showed a positive response to treatment. MRI of the cervical, thoracic, and lumbar spine showed overall improvement of metastatic disease with complete resolution of the epidural disease in the thoracic spine. MRI of the pelvis showed signs of inflammation but no evidence of new or progressive metastatic disease (Figure 8). PET/CT showed significant improvement in FDG avid lesions of the axilla and appendicular skeleton.

**FIGURE 9:**
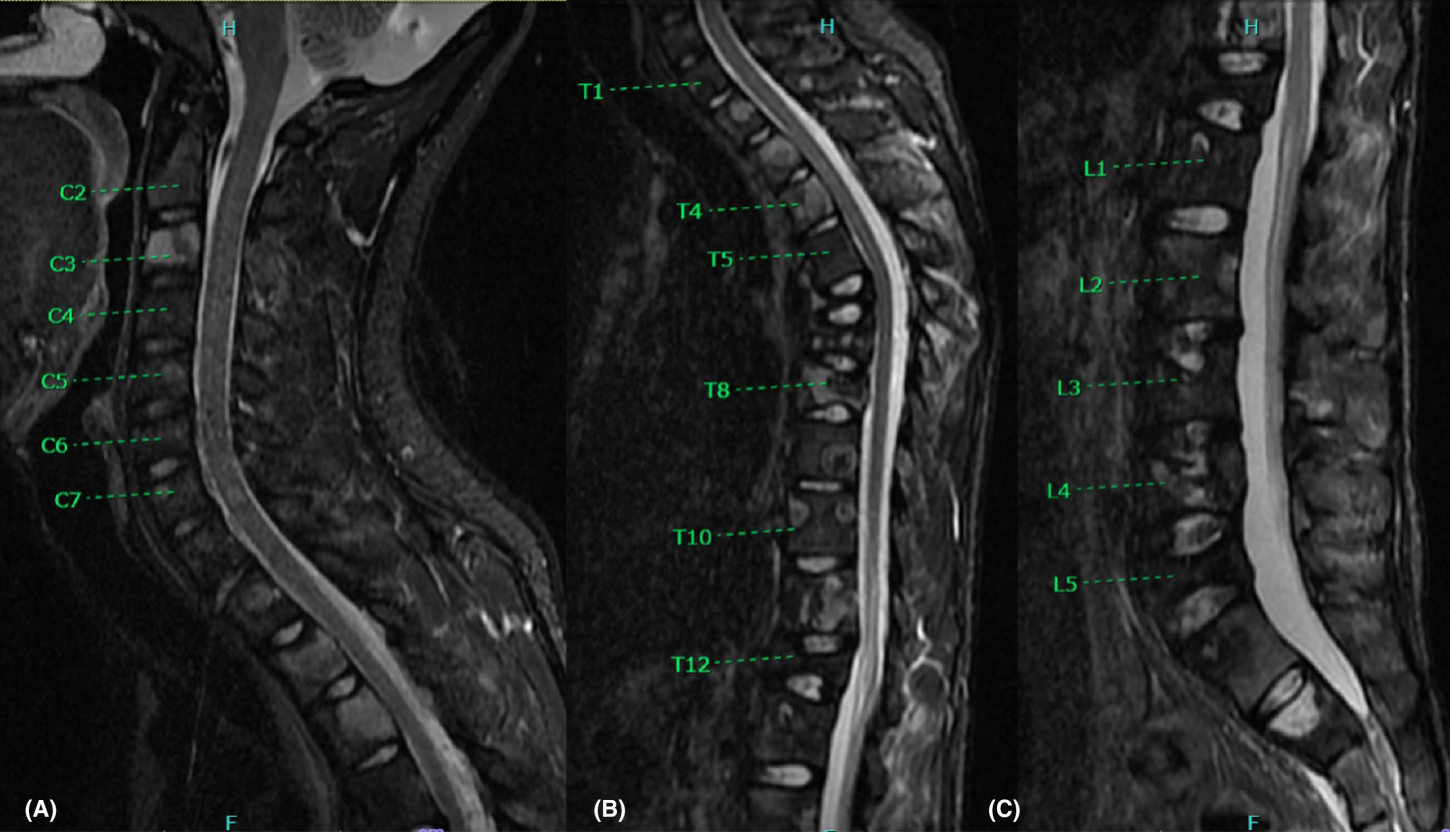
Post‐treatment MRI, including mid‐sagittal inverse recovery images of the cervical (A), thoracic (B) and lumbar spine (C) at 14 months after initiating treatment. This imaging demonstrated multifocal extensive osseous metastatic disease through the cervical and thoracic spine with multiple lesions, and multifocal osseous metastatic disease throughout the lumbar spine, which is slightly improved compared to prior MRI at 10 months (Figure 8). Once again, the osseous metastatic lesions appear reduced in size compared to the pre‐treatment MR images.

MRI imaging 14 months after initiating treatment demonstrated multifocal osseous metastatic disease through the cervical and thoracic spine with multiple lesions, and multifocal osseous metastatic disease throughout the lumbar spine, which was stable compared with prior MRI from February of 2022 (Figure 9). Follow‐up with oncology 1 year after the onset of chemotherapy and 8 months after initial radiation therapy reveals the patient is doing well with minimal pain and no neurological complaints, or dysfunction. He underwent subsequent radiation therapy to residual sites for 3 weeks, which was completed 8 months after his initial evaluation. He also completed several cycles of oral pazopanib. The patient reported an Oswestry Disability Index (ODI) score of 14 1 year after initiating treatment, which is consistent with mild disability.

## 3 DISCUSSION

**FIGURE 10:**
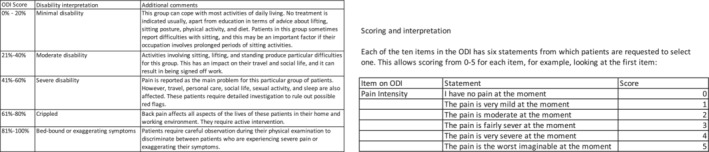
(A) Oswestry Disability Index (ODI) is a patient‐reported outcome that assesses low back pain in the hospital setting on a range from 0 to 50, correlating with a range from no disability to completely disabled. (B) The score is based on 10 questions regarding pain intensity, personal care, lifting, walking, sitting, standing, sleeping, sex life, social life, and traveling.^16^ The subject of this case report reported a post‐treatment ODI score of 14 at 13 months after initiating treatment, which is consistent with minimal disability.

In this report, we present a case of stage IV metastatic MCS in an adolescent male involving diffuse regions of the thoracolumbar spine and pelvis. Imaging 1 month after the completion of chemotherapy and radiation therapy showed the patient had a good response evidenced by reduction in size and irregularity of the thoracic and lumbar lesions. One year after onset of nonsurgical treatment, the patient does not have any significant axial pain, and MRI showed a reduction in size of multiple lesions without evidence of local recurrence. The patient reported an Oswestry Disability Index (ODI) score of 14 1 year after initiating treatment, which is consistent with mild disability (Figure 10).

Due to the rarity of MCS, there is a dearth of literature to guide treatment protocols. Chemotherapy is generally thought to be ineffective in treating MCS, but its role is unknown.^12^ In the absence of metastases, wide margin resection is the preferred treatment for intermediate‐ to high‐grade chondrosarcoma, with the goal of achieving an en bloc resection with negative margins.^12^ However, wide margin resection can be associated with significant morbidity, and may not always be possible, especially in spine. In the setting of metastases, a curative resection is not possible, therefore the role of a significant surgical reconstruction must be carefully considered and weighed against the potential morbidity.^12^

Despite the conventional view that MCS is non‐responsive to chemotherapy, some authors have suggested that MCS, in contrast to other chondrosarcoma subtypes, may be susceptible to chemotherapy due to its composition of a high proportion of small cells.^3^, ^5^ The preferred chemotherapy regimens described in the literature for MCS are typically those used to treat Ewing's Sarcoma. Nevertheless, the overall efficacy of chemotherapy in MCS is unknown.^3^, ^12^ There exists some small case series of patients presenting with metastatic MCS who were not deemed eligible for surgery and were thus treated with combination chemotherapy and radiotherapy resulting in favorable survivorship in these subjects of these small series. One study by Harwood et al.^13^ found that three out of five patients with metastatic MCS who were treated with concurrent chemotherapy and radiotherapy and did not undergo surgical resection, demonstrated progression‐free survival for 12 months or longer. There is a lack of research regarding which chemotherapy regimen may be best for MCS, but MCS may be responsive to doxorubicin chemotherapy, and agent that has been shown to be effective in Ewing's sarcoma.

It has been suggested in the literature that MCS, similar to other chondrosarcoma subtypes, is resistant to radiotherapy since the cancer grows slowly with a low fraction of dividing cells.^12^ Therefore, radiation therapy for chondrosarcoma is utilized for local control, palliative reasons, or after incomplete resection. However, MCS has been observed to be a more radiosensitive subtype.^12^ Conventional radiotherapy with photons is limited as the tumors are usually less accessible due to their proximity to delicate neurological and visceral structures. In contrast, particle therapy, like proton beam radiation, is advantageous as it allows for a minimal exit dose and therefore spares critical structures adjacent to the tumor.^12^

The patient also underwent multiple cycles of immunotherapy with pazopanib. Pazopanib is a targeted molecular therapy that inhibits tyrosine kinase. It was originally created for treatment of advanced renal cell carcinoma (RCC), but a randomized controlled trial among 372 patients showed better progression‐free survival for patients with metastatic non‐adipocytic soft tissue sarcoma after previous chemotherapy.^14^ However, the improved progression‐free survival did not correlate with a mortality benefit. Furthermore, the role of pazopanib in treating MCS is unproven.

## 4 CONCLUSION

Mesenchymal chondrosarcoma is an aggressive, malignant neoplasm that has historically been treated with surgical resection when feasible. In addition, whether treated surgically or nonsurgically, local recurrence is a feared complication of MCS, although the rates of local recurrence for MCS are unknown. There is limited evidence supporting use of radiation therapy and chemotherapy in patients with MCS. However, in metastatic MCS, these modalities may prevent local recurrence and may prolong survival. Further research regarding the role of chemotherapy and radiotherapy for MCS is warranted to further characterize its efficacy.

## AUTHOR CONTRIBUTIONS

**Leeann Qubain:** Data curation; formal analysis; visualization; writing – original draft; writing – review and editing. **Brandon P. Hirsch:** Conceptualization; data curation. **Naresh Reddivalla:** Data curation; validation. **Ali A. Baaj:** Validation. **Lee R. Leddy:** Validation; writing – review and editing. **Robert A. Ravinsky:** Conceptualization; project administration; visualization; writing – review and editing.

## FUNDING INFORMATION

No external funding was received for the research and publication of this article.

## CONFLICT OF INTEREST

All authors declare that they have no conflicts of interest.

## CONSENT

Written informed consent was obtained from the patient to publish this report in accordance with the journal's patient consent policy.

## Data Availability

Due to the nature of this article as a case report, there is no external data available for readers to access. All clinical information was taken from chart review at Banner Hospital and cannot be released due to HIPAA regulations.
